# Forecasting Sales Profiles of Products in an Exceptional Context: COVID-19 Pandemic

**DOI:** 10.1007/s44196-022-00161-x

**Published:** 2022-11-18

**Authors:** Rita Sleiman, Ahmad Mazyad, Moez Hamad, Kim-Phuc Tran, Sébastien Thomassey

**Affiliations:** 1 University of Lille, ENSAIT, GEMTEX-Laboratoire de Génie et Matériaux Textiles, 59000 Lille, France; 2 DRIVEN, 59510 Hem, France

**Keywords:** Demand forecasting, Sales profiles, K-means clustering, Random forest, Sales profiles correction

## Abstract

Accurate demand forecasting has always been essential for retailers in order to be able to survive in the highly competitive, volatile modern market. However, anticipating product demand is an extremely difficult task in the context of short product life cycles in which consumer demand is influenced by many heterogeneous variables. During the COVID-19 pandemic in particular, with all its related new constraints, the fashion industry has seen a huge decline in sales, which makes it difficult for existing sales forecasting methods to accurately predict new product sales. This paper proposes an original sales forecasting framework capable of considering the effect of the COVID-19 related crisis on sales. The proposed framework combines clustering, classification, and regression. The main goals of this framework are (1) to predict a sales pattern for each item based on its attributes and (2) to correct it by modelling the impact of the crisis on sales. We evaluate our proposed framework using a real-world dataset of a French fashion retailer with Omnichannel sales. Despite the fact that during the lockdown period online sales were still possible, consumer purchases were significantly impacted by this crisis. Experimental analysis show that our methodology learns the impact of the crisis on consumer behavior from online sales, and then, adapts the sales forecasts already obtained.

## Introduction

In the last decade, the interest in sales forecasting of new products has been steadily rising in multiple domains such as electronics [1], food [2, 3], automobiles [4], computer products [5], and fashion [6–8]. Sales forecasting is crucial for supply chain management and inventory planning, and more especially in fluctuating and complex markets such as the fashion sector. The fashion industry is faced with both stockout and overstock caused by the long production and supply lead times and the continuous fashion trend change [9]. Hence, in order to improve profitability as well as customer satisfaction in a very competitive sector, the development of efficient and proper sales forecasting systems is required [10, 11].

Obtaining accurate sales forecasts of fashion products has always been an extremely challenging task due to the fact that the customer’s demand may be affected by a wide range of factors including discounts, holidays, and weather changes. In addition, during the past few years, the “Fast Fashion” has revolutionized the fashion industry by introducing additional challenges such as the increase in product variety and the shortening of product life cycles, increasing dynamic, complexity, and competition of the market [12].

In the literature, academics and researchers address these constraints with sales forecasting systems based on machine learning techniques and specifically developed for the fashion industry. However, when unprecedented external factors suddenly arise, such as pandemics, political, economical, or environmental crises, purchasing behaviors are deeply impacted, and existing forecasting systems, even based on advanced computational techniques, become inefficient. In fact, most of the proposed systems in the literature require to be trained on historical sales data in order to predict sales of new products under a stable environment, namely with identical decision rules and factors influencing consumer purchasing decisions.

The COVID-19 pandemic is a typical situation where standard forecasting systems suddenly become obsolete. A heterogeneous range of health, economic, and social consequences has been experienced worldwide due to the COVID-19 pandemic [13, 14], with substantial implications for crisis management. More specifically, the lockdowns imposed in many countries have directly and strongly impacted the sales of fashion products. After the shock of the early weeks of the crisis, fashion companies have a critical need for sales forecasting models capable of taking into consideration this fluctuating situation to ensure online sales and anticipate the reopening of their stores. In order to manage their inventory and replenishment, it is crucial for fashion retailers to know what types and quantities of products consumers will be willing to buy in this new environment.

Motivated by a real business analytic project, this study proposes a new methodology for forecasting sales in the unusual and exceptional context of the COVID-19 pandemic. Despite the fact that there exist lots of sales forecasting systems proposed in the literature, to the best of our knowledge, these systems rely on learning techniques based on historical data and are thus not suitable in a such fluctuating environment. Therefore, the key contribution of this work lies in an original approach to implementing machine learning techniques in an unprecedented context such as the COVID-19 pandemic. The objective is to develop a novel sales forecasting framework for fashion retailers that can predict product sales in an environment where consumer purchasing behaviors are drastically changed due to external unusual factors, impacting the market in depth. In order to deal with this unexpected and complex real-world problem, our methodology relies on the implementation of advanced machine learning techniques from a comprehensive analysis of the crisis impact on the different types of products. For designing the proposed framework, key issues are addressed in three steps: (1) a sales profile of each product is defined from its characteristics, using K-means clustering and Random Forest (RF) classification, (2) the impact of the pandemic on the consumers’ behaviors is analyzed and modeled, (3) new sales forecasts are computed with a regression model taking into consideration the impact of the pandemic and the sales profiles.

The rest of this paper is structured as follows. Section 2 presents a literature review on the existing sales forecasting systems. A description of the problem, as well as the motivation for this work, are presented in Sect. 3. Section 4 describes the proposed methodology in detail along with a short review of the Machine Learning (ML) algorithms that were used. The experimental setup and the obtained results are presented in Sects. 5 and 6, respectively. The conclusion is provided in the last section.

## Literature Review

Several forecasting models have been proposed in the literature starting from the classical methods, such as exponential smoothing and regression models, to more advanced ones, like neural networks and hybrid methods [15]. However, applying such models in the fashion market faces a critical challenge which is the lack of historical data. In fact, sales forecasting of fashion products can be done at one of the two levels: sales forecasting per family of products, or sales forecasting at the Stock-Keeping Unit (SKU) level (aka sales forecasting per product). In the case of forecasting at a family of products level, data aggregation (aggregation of sales for products belonging to the same family of products) is required in order to get historical data of several years, and the forecasting problem can be dealt with as a time series forecasting problem. However, this strategy cannot provide sales forecasting at the SKU level. Hence, models based on analogous forecasting are generally used to overcome this problem [16, 17]. Such models assume that similar products may have the same sales behavior. By finding patterns and links between sales and descriptive criteria, these methods allow forecasting sales of new products with higher accuracy without the need of having historical data.

A preceding study done by Chang and Lai [18] suggests combining a Self Organizing Map with the Case-Based Reasoning (CBR) method in order to generate sales forecasts for newly released books. Their method gives more accurate forecasts than traditional CBR. Hadavandi et al. [19] propose a sales forecasting approach for forecasting monthly printed circuit board sales by integrating genetic fuzzy systems and the K-means data clustering technique. The experimental results show that the proposed approach yields better results compared to other approaches. Thomassey and Fiordaliso [20] construct a sales forecasting system for mid-term forecasting of fashion products. It is a framework that combines clustering and C4.5 decision tree classifier to group the sales profiles of existing items into different patterns, and then assign a matching demand pattern for each new item based on its corresponding descriptive criteria. They validate their approach using real sales data and show that the proposed model outperforms other existing forecasting systems. A similar approach is applied by Chae et al. that uses a hybrid model of clustering and decision tree to forecast sales patterns of new products [21]. Another study conducted by Thomassey and Happiette [22] applies the same approach using a SOM for clustering and a Probabilistic Neural Network (PNN) for classification. Results show that PNN could not outperform all other classification methods used for comparison. Lu and Wang [23] use independent component analysis, a growing hierarchical SOM, and support vector regression to build a sales forecasting system for computer wholesalers. Experimental results show that the presented model could accurately forecast sales. Another sales forecasting system of fashion products is proposed by Fallah Tehrani and Ahrens [24] which uses a probabilistic approach to classify fashion items in terms of sales, followed by a kernel machine approach to predict the number of sales. By applying their approach to real data from an apparel retailer in Germany, they obtained more robust and reliable results. An accurate and scalable sales forecasting tool for new products is also developed by Baardman et al. [25]. It considers clustering the products into groups using multinomial logistic regression based on each item’s attributes. Thereafter, multiple linear regression, Support Vector Machine (SVM), and neural networks are used to generate sales forecasts for each cluster. Their evaluations based on real-world data give promising and robust results over different categories and industries. A deep learning approach is explored by Loureiro et al. [26] to forecast sales of new products in the fashion industry based on product characteristics and the opinion of domain experts. Results found that even though a deep learning model may have good prediction performance, it does not significantly outperform the RF algorithm whose training process is much more simple. Another study formulates the forecasting problem as a classification task in which the objective is to forecast sales for new products by applying five different machine learning models—logistic regression, support vector classification, nearest neighbors, XGBoost, and multi-layer perceptron. Results show that the XGBoost model outperforms the other models in all the evaluation metrics [27]. Shanshan proposes a new hybrid model to forecast the demand for new products and applies it to a real application of the Body Shop. The proposed model combines a linear model that captures the effect of time-varying exogenous variables, and choice models to incorporate cannibalization with limited data [28].

The advanced techniques used in these models enable the forecasts to take into account uncertainties, missing data, etc. However, the obtained results strongly rely on the reliability of the learning data and more specifically the stability of the market environment.

## Problem Description

There have been a number of negative impacts of the COVID-19 pandemic. Firstly, the pandemic resulted in hospitals and health facilities being overwhelmed with COVID-19 patients, which has led the health system, with restricted capacity, obliged to give priority to the more vulnerable patients such as the elderly, women and children [14]. Secondly, The global pandemic of COVID-19 has adversely impacted business worldwide. The constraints imposed by the COVID-19 pandemic had affected consumers’ buying behavior, resulting in a decrease in total retail sales and profits, especially for non-essential goods. Moreover, start-up businesses were also negatively impacted by the pandemic which has limited their creation and threatened their survival [29]. The pandemic caused many job losses as well; for instance, over 125000 retail agents lost their jobs in the United Kingdom [30]. However, in spite of the large negative impact of COVID-19 on the retail sector, some positive benefits had also been noted. A positive outcome of the pandemic has been the implementation of innovative marketing strategies by retailers. In fact, one way the pandemic has benefited retailers is that it has prompted them to innovate and rethink their strategies and priorities [31]. As stated by Matthew Shay, President and CEO of the National Retail Federation, the challenges faced during this difficult period demonstrated the resilience of retailers, as well as their adaptability to implement changes to improve the consumer experience. For instance, by using their physical stores, retailers have expanded the availability of online purchasing, pick-up in-store, curbside delivery, and ship-from-store options, thereby enhancing their supply chain efficiency and delivering more goods to consumers within shorter timelines [32].

In this study, we address a real sales forecasting problem of a French fashion retailer, in a context of a sudden and unforeseen event that makes the traditional and existing forecasting systems unusable. In fact, the fashion industry was one of the most negatively impacted industries by the COVID-19 pandemic in the economic sector [33, 34]. Comparing the second quarter of 2020 with the same period in 2019, the garment sector in Europe experienced a 37.4% drop in production. And among all retail sales, clothing products saw the greatest decline, going down 43.5% [35]. Consumer purchase behavior was changed due to several factors. First, the strict measures taken by the government to prevent the spread of the virus, such as the strict lockdown, the stores’ closures, and the events’ cancellations, have hugely impacted fashion brands by having zero sales in stores for the first time ever. Studies have shown that the change in consumers’ behavior included changes in mindset, brand loyalty, and price sensitivity, changes in prioritization, and changes in shopping methods [36]. In fact, as the pandemic has affected people’s ability to socialize in the same way as before, they are less likely to purchase luxury fashion items during this time. Instead, it’s common for people to spend more on casual clothing and activewear since they wear these things on a regular basis. Moreover, consumers have become more price sensitive as a result of increased unemployment. Hence, at the end of the lockdown, fashion retailers were left in a hard situation where they had to rapidly act to face the totally new challenges caused by the pandemic.

Meanwhile, due to the resulting gap in the historical data, as well as the huge change in consumer behavior, existing sales forecasting systems could no longer be applicable. Consequently, retailers were in urgent need of new forecasting methodologies so they can save as much as possible of the unsold previous collection, and create a new collection that best fits the actual consumers’ demand. Therefore, this paper presents a new sales forecasting methodology developed to predict sales of fashion products taking into account COVID-19’s pandemic impact on sales. In fact, the proposed methodology can also be generalized to other domains and other situations where sales are severely disrupted for a period of time making existing forecasting models ineffective.

**Fig. 1:**
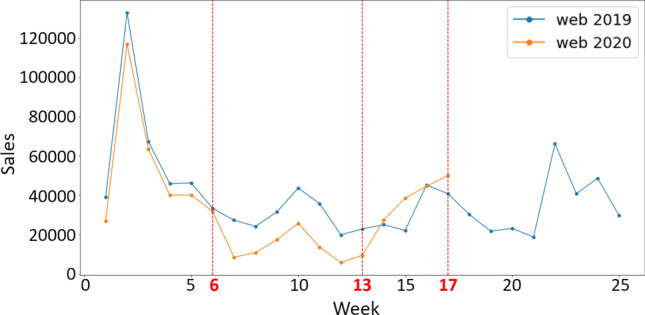
Online sales comparison between years 2019 and 2020

Even though online sales were still possible during the lockdown period, consumers’ online purchases were also deeply impacted by this crisis. Figure 1 shows the difference between weeks’ sales of the first half of 2019 (normal situation) and 2020 (with COVID-19) for web sales. Being in the (18th) week of 2020, only the sales until the (17th) week were available. In fact, the beginning of the pandemic’s impact on sales in France started at week 6, and the lockdown ended at the beginning of the (20th) week, which was announced two weeks earlier. Thus, at week 18, it was crucial for retailers to revise their forecasts for the end of the lockdown (week 20) to ensure their economic survival. When analyzing the sales patterns presented, it can be noticed that the period between weeks 1 and 17 is essentially divided into 3 parts, each with a different purchasing behavior, as follows:

- Weeks 1–6: normal purchasing behavior (before the crisis began),
- Weeks 6–13: the full impact of the changes (huge drop in sales quantity with the beginning of the lockdown),
- Weeks 13–17: resilience of consumer behavior (steady increase in sales quantities).

The huge drop in sales quantity mentioned above (weeks 6–13) can be explained by a rational behavior faced when an unexpected and unknown situation occurred. When the lockdown was announced, consumers were panicking and all of a sudden they focused on buying only essential goods in bulk (ex. flour, oil, toilet papers, etc.) [37, 38]. Such behavior was also noticed during previous epidemics such as the SARS outbreak, Fukushima disaster, MERS outbreak, etc. as mentioned in [39], and known as Economic Elastic Behavior. With the lockdown announcement, fashion retail sales suddenly stopped, and as the consumer panic subsides, the market starts recovering, which explains the two-stage evolution of the sales between the weeks 6 and 17 illustrated in Fig. 1: weeks 6–13, and 13–17.

In this disturbed environment, the main issue for fashion retailers was to know what products and what quantities can still be sold after the lockdown, namely from week 18. Thus, in order to predict the sales after the lockdown, we developed a specific forecasting model which relied on online sales data and more especially on sales during the recovery period (weeks $> 13$).

## Methodology

**Fig. 2:**
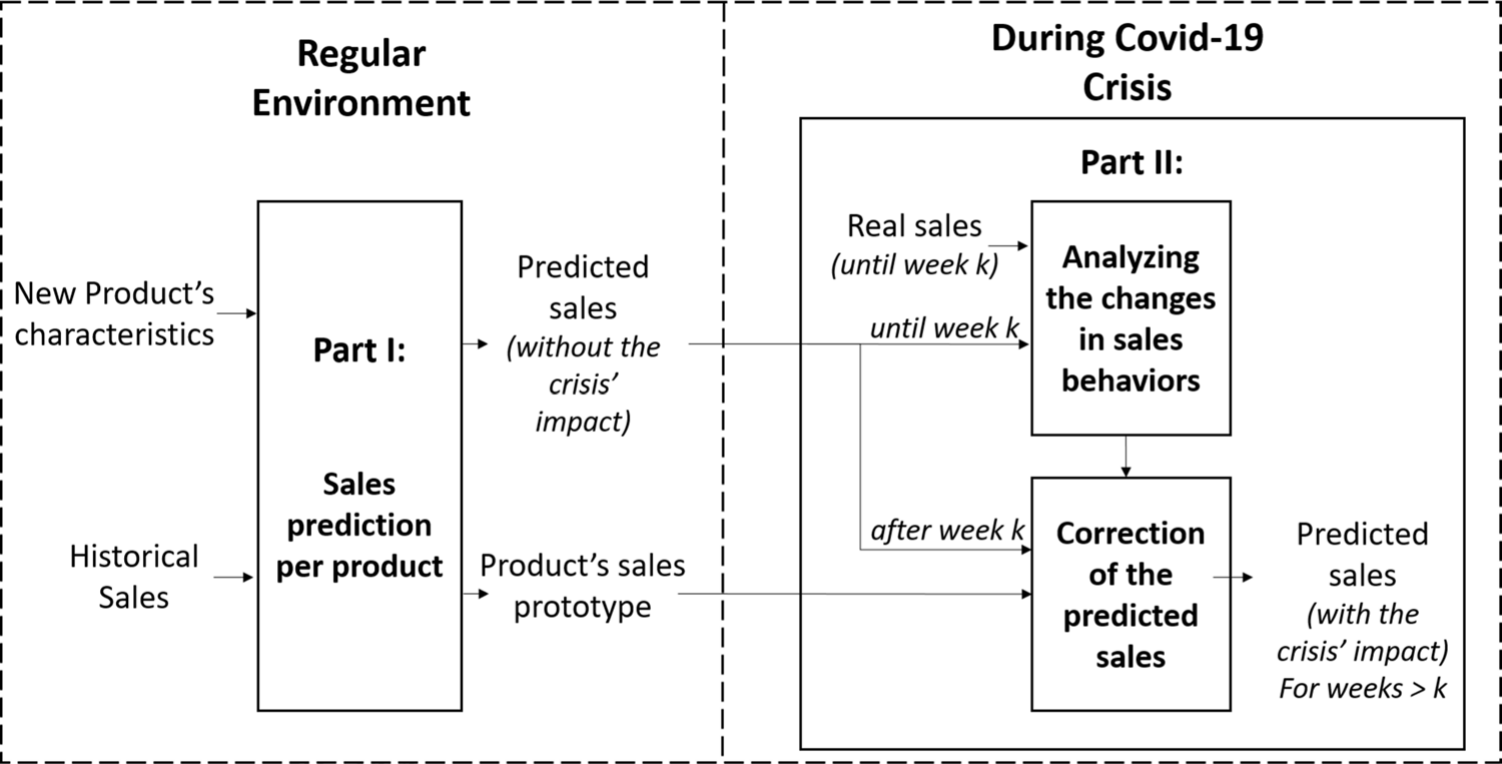
Overview of the proposed methodology

In this section, our proposed sales forecasting framework will be presented in detail. By combining several ML algorithms: K-means for clustering, RF for classification, and Polynomial Regression for forecasts’ correction, we propose a forecasting model capable of predicting a sales profile for each item taking into account the Covid-19’s crisis effect on sales. This system is mainly composed of two parts, as shown in Fig. 2. In the first part, a forecasting methodology inspired by the work of Thomassey and Fiordaliso [20] was applied in order to generate sales forecasts for each new item based on its characteristics using clustering and classification techniques. It was already demonstrated that this methodology is capable of predicting sales in a regular situation [17, 20]; using historical sales of similar items, along with the characteristics of the new product, this method is capable of detecting different sales prototypes (different clusters) in historical data and assigning a corresponding sales prototype to the new products without the impact of the covid-19 crisis. The second part of the proposed methodology aimed to adjust the forecasts obtained in the first part for weeks after week *k* (*k* = 17, as detailed in Sect. 3), so they better fit the consumers’ demand during Covid-19’s pandemic. This was done using a systematic approach by product clusters during the purchase recovery period previously identified; using the obtained sales forecasts from part I along with the real sales until week k, this part corrects the forecasted sales by adding the impact of the crisis. The correction model used in this part is specific to the corresponding sales prototype of the considered new product, as obtained from part I. In the following parts, a quick review of K-means and RF is presented in Sects. 4.1 and 4.2, respectively. Then, the proposed forecasting system is detailed in Sect. 4.3.

### K-Means Clustering

K-means algorithm is an unsupervised non-hierarchical clustering algorithm commonly used in ML applications [40]. Given initially an integer K, K-means allows dividing the observations in the dataset into K distinct groups, called clusters. Thus similar data will be found in the same cluster. This is done by minimizing the sum of the squares of the distances between each observation and the mean of its corresponding cluster.

However, choosing the number of clusters K is a very critical task, especially when no prior assumptions about the data exist [40]. A large number can lead to an excessive partitioning of the dataset which may prevent the discovery of interesting patterns. On the other hand, a number of clusters that is too small will potentially lead to clusters that are too generalized. Therefore, the difficulty lies in choosing an appropriate clusters’ number K that allows highlighting interesting patterns in the dataset. To do so, there are several methods that can be used such as the Elbow Method [41] and choosing K based on the Silhouette Coefficient [42]. In this study, we applied a tuning procedure based on the overall forecasting error in order to choose the optimal number of clusters (see Sect. 4.3.2).

### Random Forest Classification

The RF algorithm is a frequently used ML algorithm proposed by Leo Breiman in 2001 [43]. It allows performing classification as well as regression tasks by conducting parallel learning on a set of randomly built decision trees. The optimal number of trees is an important parameter that has to be initially chosen, which can be several hundred or more. Concretely, each tree in the RF is trained on a random subset of data according to the principle of bagging, with a random subset of features (inputs to the model), which allows obtaining an efficient and robust model [43, 44]. The predictions are then averaged in the case of regression trees or used for a vote in the case of classification trees. The RF algorithm is known to be one of the most efficient and applied classifiers for the fact that it can directly handle categorical data and missing values without the need for previous data preprocessing [45].

**Fig. 3:**
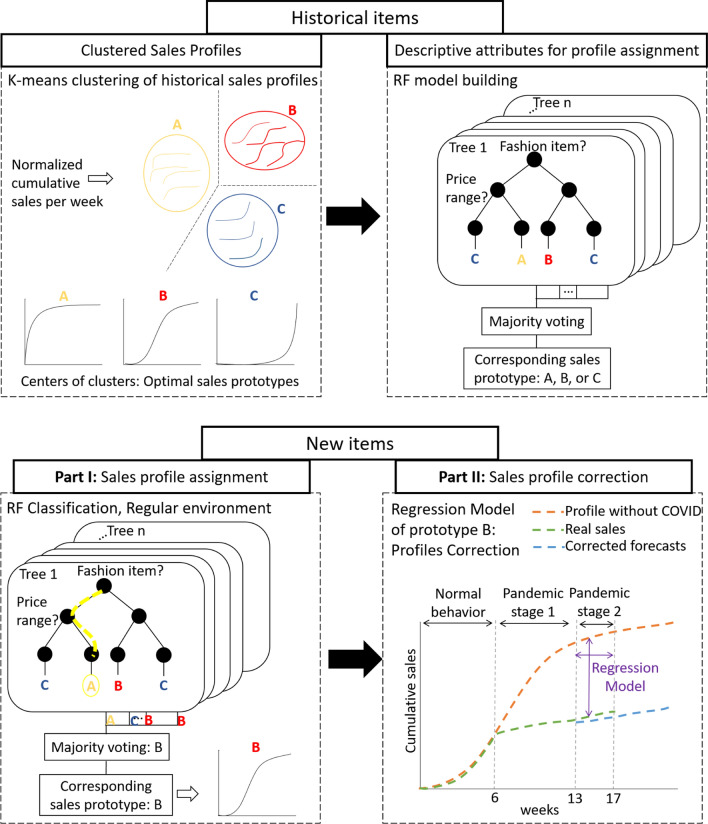
Schematic representation of the proposed sales forecasting system

### Proposed Solution Methodology

The proposed methodology starts with a mid-term sales forecasting system based on regular historical data (namely in a quite stable market environment, as if there was no sanitary crisis) which provides the sales profile of each item. Then, a corrective model is defined from the comparison and analysis of online sales before and during the lockdown. Our methodology represented schematically in Fig. 3 uses data of all items belonging to the same family of products. For the historical data, it assumes that both products’ characteristics and sales data are available. The following parts present the details of the proposed methodology.

#### Historical Sales Profiles

The first step of the forecasting system is the data preparation which consists of obtaining the sales profile for each of the historical items. The sales profile of a certain product represents the pace with which this product will be sold during a certain period of time; it gives an insight of the consumer demand for such a product in this period. In this study, a fixed forecast horizon of 23 weeks corresponding to the spring-summer collection for fashion products (January–July) is considered. Hence, for each item, calculating the sales profile requires the two following pre-processing steps:

- Normalizing the sales volume: This step is required to enable a consistent comparison of sales profiles. Let $S^i=[s_1^i,\ldots ,s_L^i]$ be the vector of length L representing the sales volume per week of product i between January and July, the normalized sales $S_n^i$ is $S^i$ divided by the total sum of the sales:
  1 $$\begin{aligned} S_n^i=\frac{S^i}{\sum _{i=1}^{L} s_l^i} \end{aligned}$$
- Calculating the cumulative normalized sales $S^i_{cn}$: The cumulative representation enables the clustering process to be more robust and straightforward than using regular sale profiles. Thus, a simple clustering technique, such as K-means, could easily identify optimal sales prototypes (see Sect. 5.2).

#### Sales Profiles Prediction

Once all the cumulative sales profiles of the historical items are obtained, the clustering method, k-means, is applied to the profiles of each family of products in order to group them into different clusters. Thereafter, the second step was to train a RF classification model capable of predicting for each new item which group of profiles it belongs to, using the corresponding features as inputs, such as its price range, its material, etc. However, the efficiency of the RF classifier, as well as the overall forecasting system, strongly depends on the number of clusters k initially chosen when applying the k-means at the first stage.

**Fig. 4:**
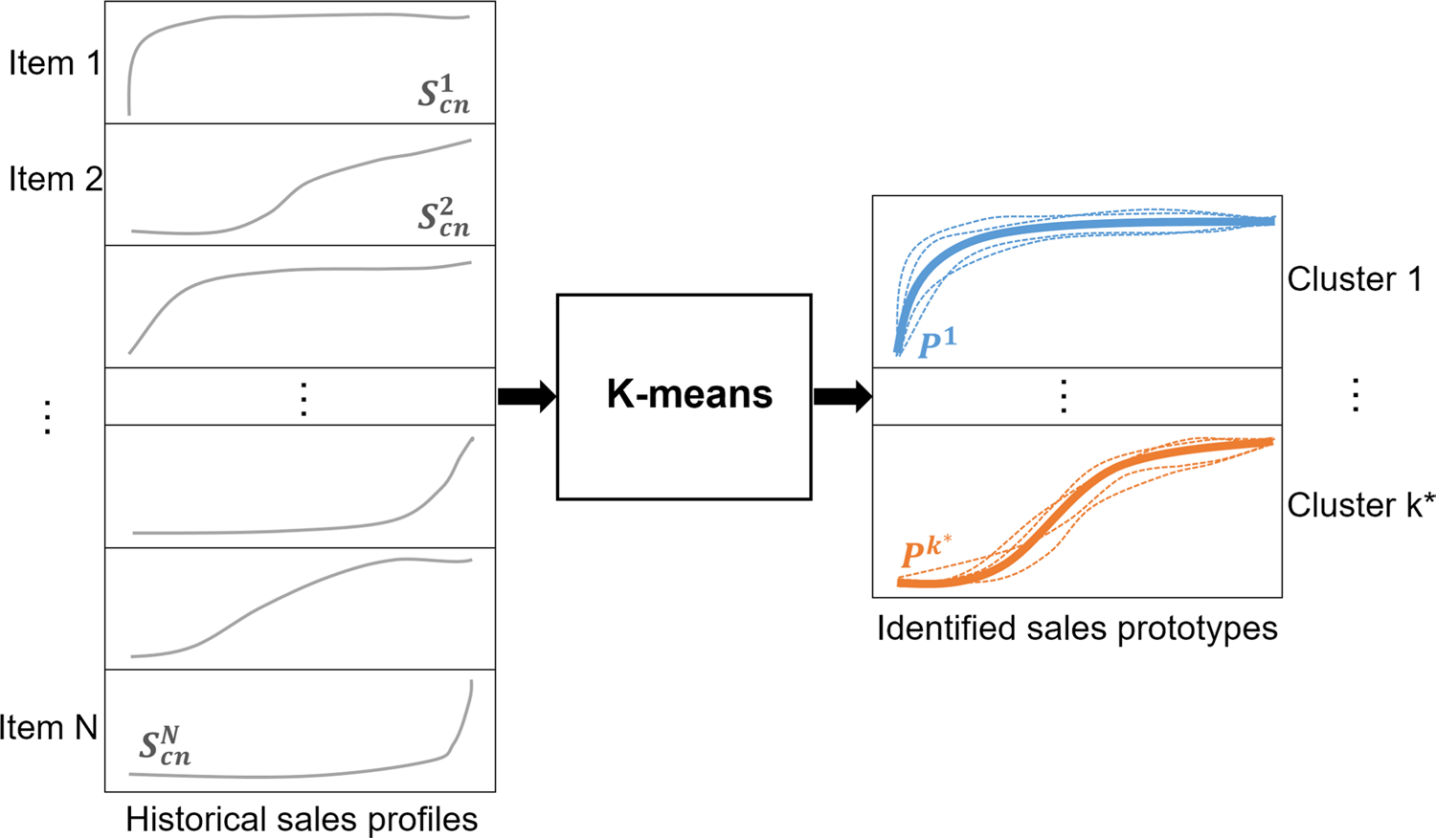
K-means clustering of sales profiles

**Fig. 5:**
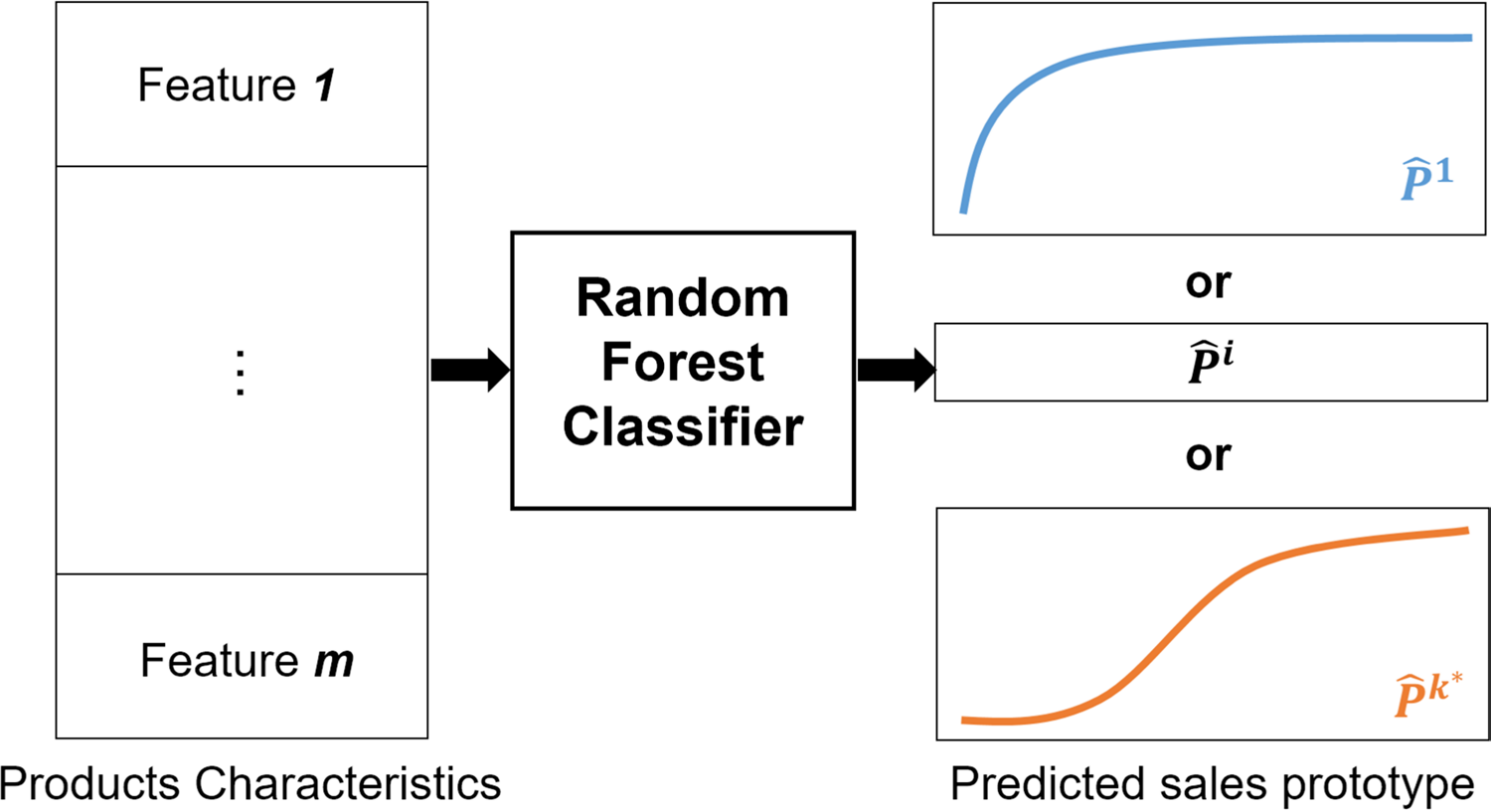
Random forest classification

**a) Tuning the number of clusters:** Although there exist several methods for identifying the optimal number of clusters for k-means (Sect. 4.1), we proceeded with a tuning procedure that aims to identify the optimal number of clusters based on the forecasting error obtained after the classification stage. This procedure ideally links the clustering and the classification stages for building an accurate sales forecasting system. We proceeded as follows:

- Selecting a number of clusters $k^*$ which varies between 3 and 7 (Based on experts’ opinion, grouping sales profiles into more than 7 clusters is not necessary for the desired objective),
- Applying k-means clustering on the N historical sales profiles with $k=k^*$ in order to identify $k^*$ different sales prototypes (Fig. 4),
- Training a RF Classifier which maps each item to its corresponding sales prototype from the $k^*$ sales prototypes already obtained, based on its different characteristics (Fig. 5),
- Calculating the mean forecasting error as the mean of the Root Mean Square Error (RMSE) between actual cumulative sales $S_{cn}^i$ and the corresponding predicted prototype ${\hat{P}}^i$ using the following formula:
  2 $$\begin{aligned} {\text {RMSE}}=\sqrt{\frac{\sum _{i=1}^{N} \left( S_{cn}^i - {\hat{P}}^i\right) ^2 }{N}} \end{aligned}$$
  where *N* is the total number of historical products,
- Choosing the optimal number of clusters $K_{{\text {optimal}}}$ that have yielded the lowest forecasting error.

**b) Sales profiles classification: ** Once the optimal number of clusters $K_{{\text {optimal}}}$ was determined, k-means was applied in order to determine the $K_{{\text {optimal}}}$ sales prototypes that best summarize historical products’ sales behaviors. Thereafter, using historical products’ data (sales and characteristics), a RF Classifier is built to model relations and patterns between a product’s characteristics and its corresponding sales prototype. Thus, the learned RF classifier could predict the corresponding sales prototype ${\hat{P}}^i$ for each new product i using the product’s characteristics as inputs. Moreover, in order to avoid the overfitting problem, 10-fold cross-validation was used during the learning of the classifier.

#### Sales Profiles Correction

At this stage, we were able to predict a sales prototype ${\hat{P}}^i$ for each new item i in 2020’s collection. However, the consumer’s behavior in 2020 was hardly impacted by the lockdown due to the COVID-19 pandemic, which has added a new unseen condition to the forecasting system trained on 2019’s historical data. Thus, sales forecasting during this disturbed period requires accurate modeling of the impact of the lockdown on sales, which could be done by taking into account the changes in consumers’ behavior during that period.

Based on the analysis performed in Sect. 3, we have identified two different purchasing behaviors for online sales after the beginning of the lockdown: the first corresponds to a state of shock during the weeks 6–13, and the second is a purchase recovery period between weeks 13 and 17. Hence, a sales corrective model was constructed, for each of the $K_{{\text {optimal}}}$ clusters of sales profiles, to correct sales for weeks after week 17. Each of the corrective models is based on a polynomial regression learned for each cluster separately on sales between weeks 13 and 17 of products belonging to this cluster. Thus, the final forecast obtained with our methodology integrates both:

- the initial cumulative sales forecast ${\hat{S}}^i$, obtained from the predicted prototype ${\hat{P}}^i$i and the real sales till week 6, which encompasses the sales behavior related to the features of the product,
- the specific impacts of the lockdown on the sales of products of the same cluster.

Let ${\hat{S}}^i_{13->L}=[{\hat{s}}_{13}^i,{\hat{s}}_{14}^i, \ldots ,{\hat{s}}_L^i]$ be the predicted sales profile where *L* is the total fixed forecast horizon of L weeks (L >17), $R_{13->17}^i=[r_{13}^i,r_{14}^i, \ldots ,r_{17}^i ]$ the real online sales between weeks 13 and 17, and $S_{corr}^i=[sc_{13}^i,sc_{14}^i,\ldots ,sc_L^i ]$ the corrected sales profile on the total forecast horizon. For modeling the impact of the crisis on sales and correcting the initial predictions ${\hat{S}}^i$, we proceeded by predicting a correction coefficient C that can be either multiplicative or additive. This could be done by training two independent regression models for weeks between 13 and 17 on coefficients ($R_{13->17}^i$ or ${\hat{S}}_{13->17}^i$ ) or ($R_{13->17}^i$ – ${\hat{S}}_{13->17}^i$ ) respectively, and then predicting the corresponding coefficients for weeks after 17.

We applied a (*k*th) order polynomial regression model with one variable. The corresponding formula is given by:

3 $$\begin{aligned} C^i=\beta _0^i+ \beta _1^i w+ \beta _2^i w^2+ \cdots + \beta _k^i w^k+ \epsilon \end{aligned}$$

where $C^i$ is the correction coefficient (additive or multiplicative) of ${\hat{S}}^i$ for week $w$.

For each sales prototype corresponding to a family of products, we applied a 5-cross validation for weeks between 13 and 17, followed by a tuning procedure based on the cross-validation errors in order to find which order for the polynomial regression and which coefficient type, additive or multiplicative, better fits the sales data.

The final corrected previsions for the product i would be calculated as follows:

4 $$\begin{aligned} S_{\text {corr}}^i = {\left\{ \begin{array}{ll} {\hat{S}}_{13->L}^i * C_{13->L}^i &{} \text { if}\; (C^i)\; \text {multiplicative, }\\ {\hat{S}}_{13->L}^i + C_{13->L}^i &{} \text { if}\; (C^i) \;\text {additive.} \end{array}\right. } \end{aligned}$$

## Experimental Setup

### Data Description

The sales data considered in this study were collected from Point Of Sales (POS) data provided by the IT system of a French fashion retailer. Our sales forecasting methodology is applied to four different datasets corresponding to four different types of products: Pants, Blouses, T-shirts, and Dresses. Table 1 presents the number of items for each family of products in 2019 and 2020 collections, used for training and testing our proposed methodology. As detailed in Sect. 4, the proposed forecasting system is composed of two parts:

- Part I—Clustering and classification for sales profiles predictions: this model was trained using historical data of products sold in 2019 (spring–summer 2019 collection), which were divided into “training” and “validation” sets during the learning process. Once the learning phase is done, the sales prediction system is applied to predict sales of 2020 items—the spring-summer 2020 collection.
- Part II—Regression for sales profiles correction: this model was trained using the obtained 2020 sales predictions, along with real 2020 sales for weeks between 13 and 17, per family of products and per sales prototype (a different regression model was trained for each of the 4 sales prototypes). The model was then tested by correcting 2020 sales from week 17 to week 21.

All the data used represents only online sales data since no sales have been made in stores during the lockdown period (Sect. 3).

Choosing the descriptive criteria of the products for the classification phase is a critical task to do especially when working on real data: some important characteristics may be not reliable or simply not found in the retailer’s dataset. For this study, we relied first on expert judgments in order to choose the products’ descriptive criteria that may impact the sales the most. A second selection is then applied from a quantitative analysis of the features’ importance in the classification phase. Six descriptive criteria were finally chosen as input to the classifier; those were the price range, the typology of the product (its type: essential, basic, fashion, etc.), the material, the sub-family, the year of the collection, and the week of the first sale.

**Table 1:** Number of items for each family of products in 2019 and 2020 collections

	Pants	Blouses	T-shirts	Dresses
Collection 2019	258	674	1822	448
Collection 2020	210	503	1319	244

### Data Preprocessing

**Fig. 6:**
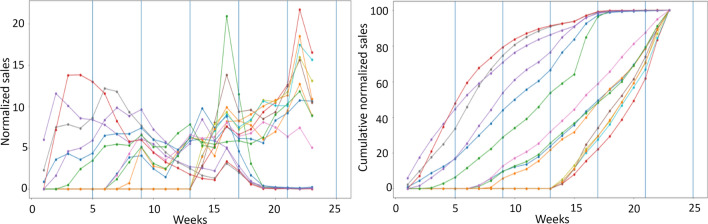
Some examples of sales profiles in the case of normalized sales (left) and cumulative normalized sales (right)

The data preprocessing is required to ensure proper implementation of our proposed methodology: only active items during the considered period were taken into consideration. Moreover, the sales volumes are normalized so that their profiles can be compared later on, as described in Sect. 4.3. However, when studying and comparing sales profiles in a given period of time (for instance spring-summer collection), we found that cumulative sales representation is significantly more suitable to understand and identify sales behavior, as illustrated in Fig. 6. Therefore, the last step of the data preprocessing consists of computing cumulative normalized sales for each item.

### Comparison with State-of-Art

For the proposed forecasting system, a direct comparison of the results with existing methodologies is difficult since, to the best of our knowledge, no prior studies were done in such a context. Thus, we proceeded by comparing forecasts, with and without correction, with real sales profiles of items belonging to the same family and sales prototype’s cluster. Moreover, in order to highlight the efficiency of the models used in the proposed system, different models are applied for comparison for the different stages—clustering and classification stages.

A comparison of different clustering techniques was necessary in order to demonstrate that a simple clustering technique implemented on cumulative sales profiles could simply, yet efficiently, identifies existing sales patterns in the data. Thus, we applied SOM followed by K-means as a more complex and advanced clustering methodology; SOM is a neural network-based unsupervised learning algorithm successfully applied to data clustering in many domains [46]. In addition, three other classification models were tested to evaluate the performance of the RF classifier: Naïve Bayes (NB) [47], SVM [48] and Multi-Layer Perceptron (MLP) [49]. The performances of the models are quantified with both the overall forecasting error and the classification accuracy as given by equations (2) and (5):

5 $$\begin{aligned} {\text {Accuracy}}=\frac{\sum _{i=1}^{N} f_n }{N} \end{aligned}$$

Such that *N* is the total number of products, and:

6 $$\begin{aligned} f_n = {\left\{ \begin{array}{ll} 0 &{} \text { if} \; {\hat{P}}^i \ne \text {actual}\, P^i, \\ 1 &{} \text { if} \; {\hat{P}}^i = \text {actual} \,P^i. \end{array}\right. } \end{aligned}$$

## Results and Discussion

As mentioned in Sect. 5.1, the methodology is applied separately on four different families of products: Pants, Dresses, T-shirts and Blouses. The proposed sales forecasting system is fully developed in python programming language which provides several open source libraries for ML applications. In this section, the results of the proposed methodology are presented in detail.

### Sales Profiles Prediction in Regular Environment

#### Sales Profiles Clustering

**Fig. 7:**
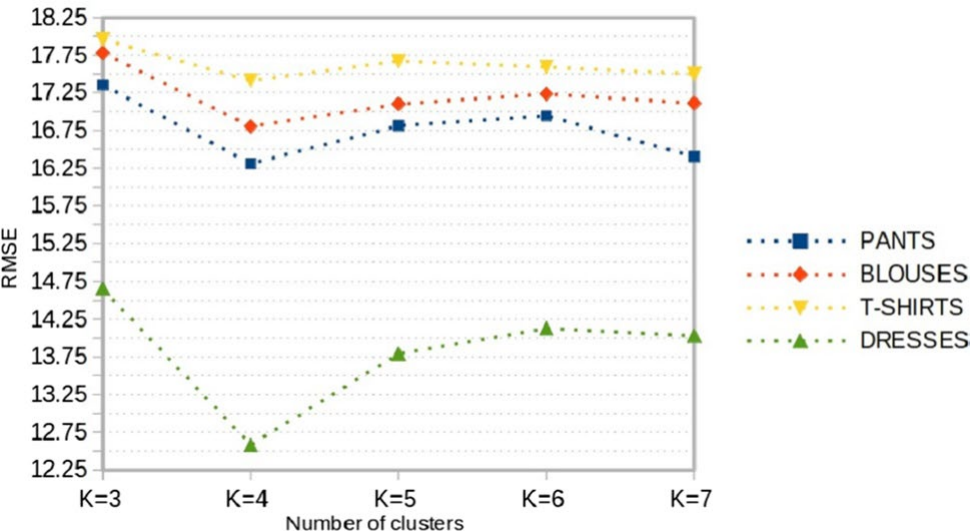
Root mean squared error obtained by cross-validation according to the number of clusters

**Fig. 8:**
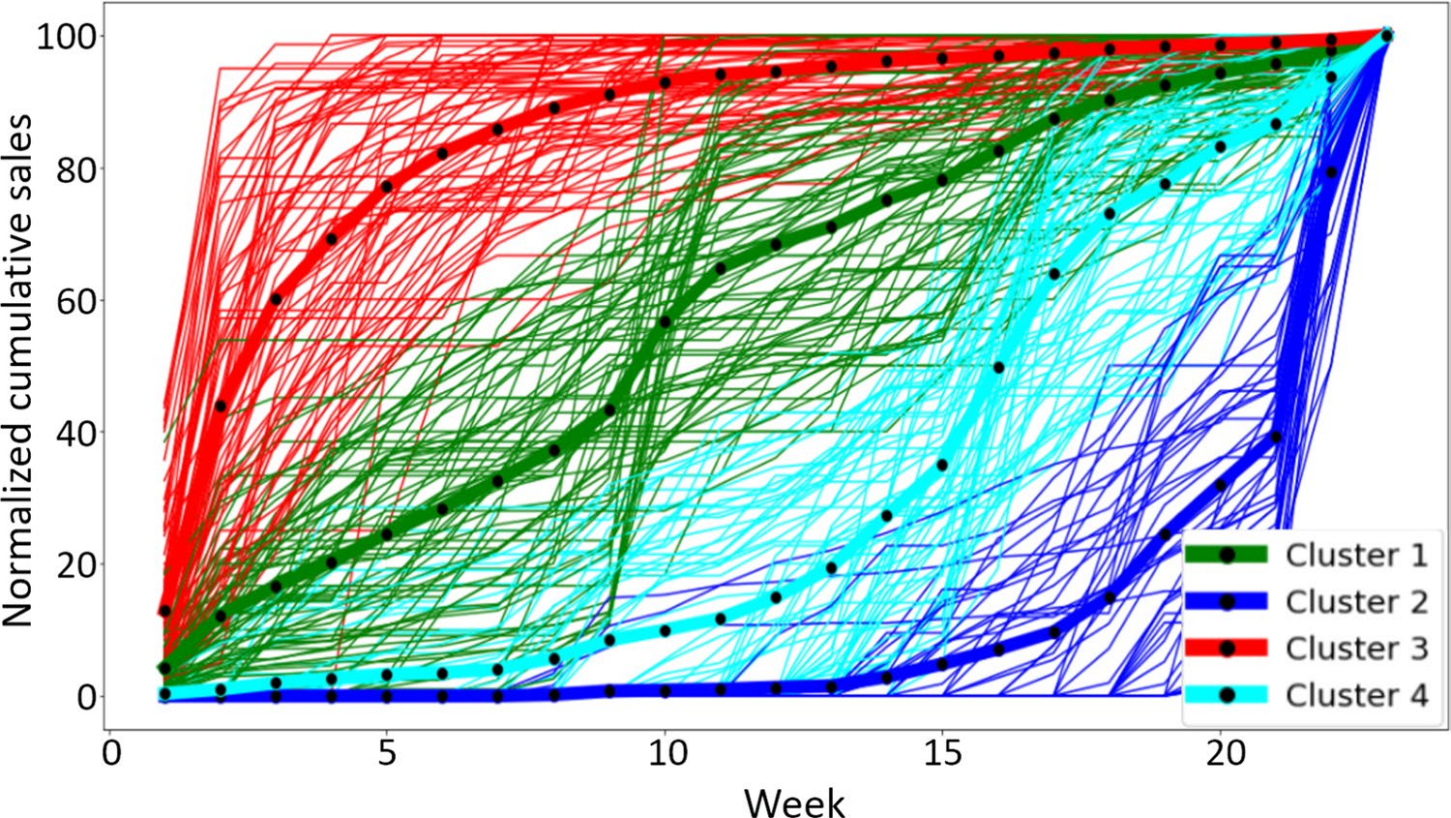
An example of the 4 clusters obtained from the sales profiles

The optimal number of clusters $K_{{\text {optimal}}}$ of sales profiles is computed for each family of products with the process described in Sect. 4.3.2. Figure 7 presents the RMSE obtained from the cross-validation data and shows that the optimal number of clusters $K_{{\text {optimal}}}$ is four for all the families of products. Thus, the sales profiles of products are summarized in 4 different patterns. Figure 8 illustrates an example of the 4 clusters of sales profiles for a family of products. The main characteristics separating these patterns are the product implementation date and its selling pace which may be influenced by the consumers’ needs and demands at a certain time. The four clusters illustrated in Fig. 8 are composed of:

- Cluster 1: products with steady sales throughout the season,
- Cluster 2: late season products, 80% of their sales are achieved in the 5–8 last weeks of the season,
- Cluster 3: early season products, 80% of their sales are achieved between weeks 5 and 10,
- Cluster 4: products with sales throughout the season but with an increasing pace from the middle of season.

**Fig. 9:**
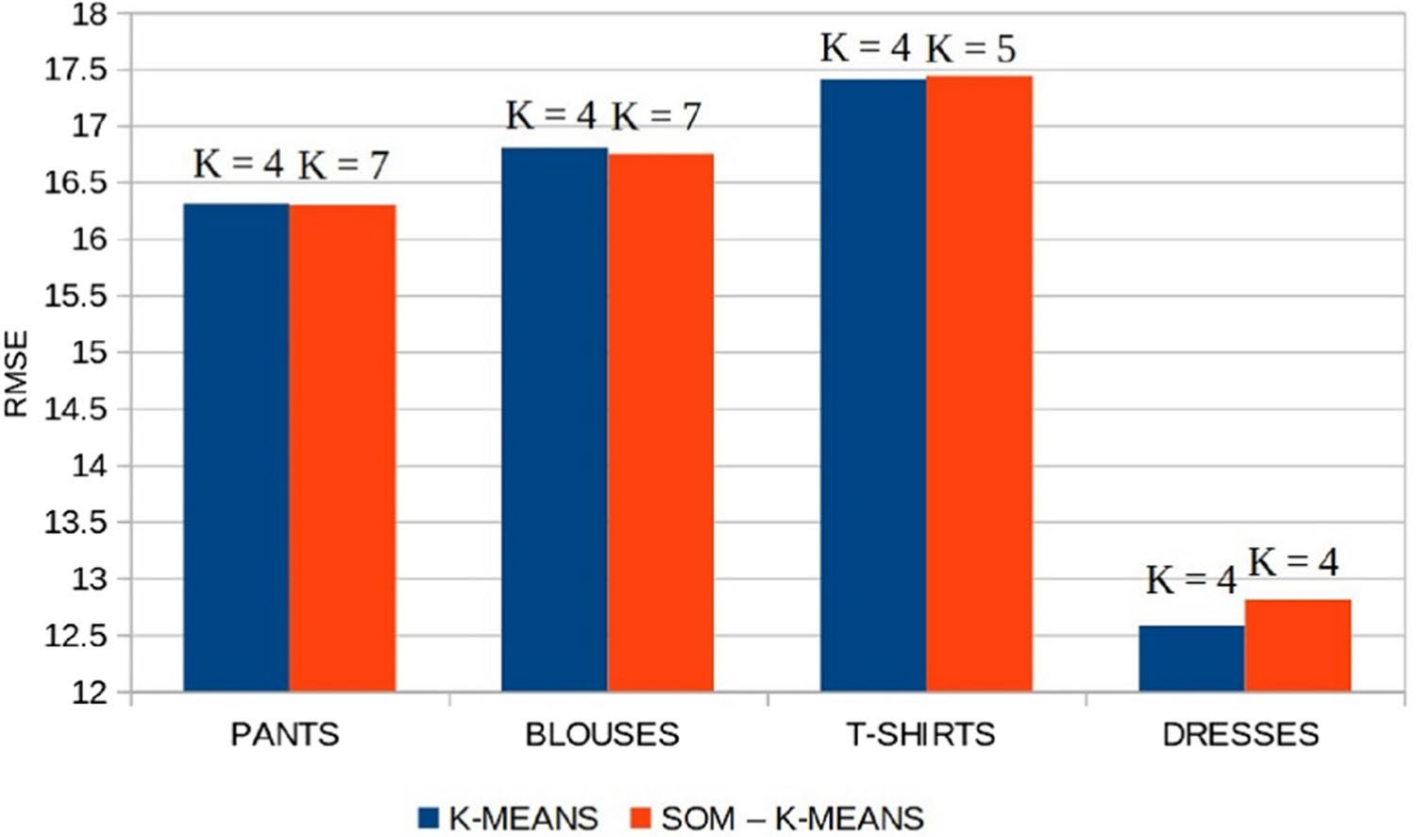
Root mean square errors when using K-means and SOM-K-means as clustering methods

**Fig. 10:**
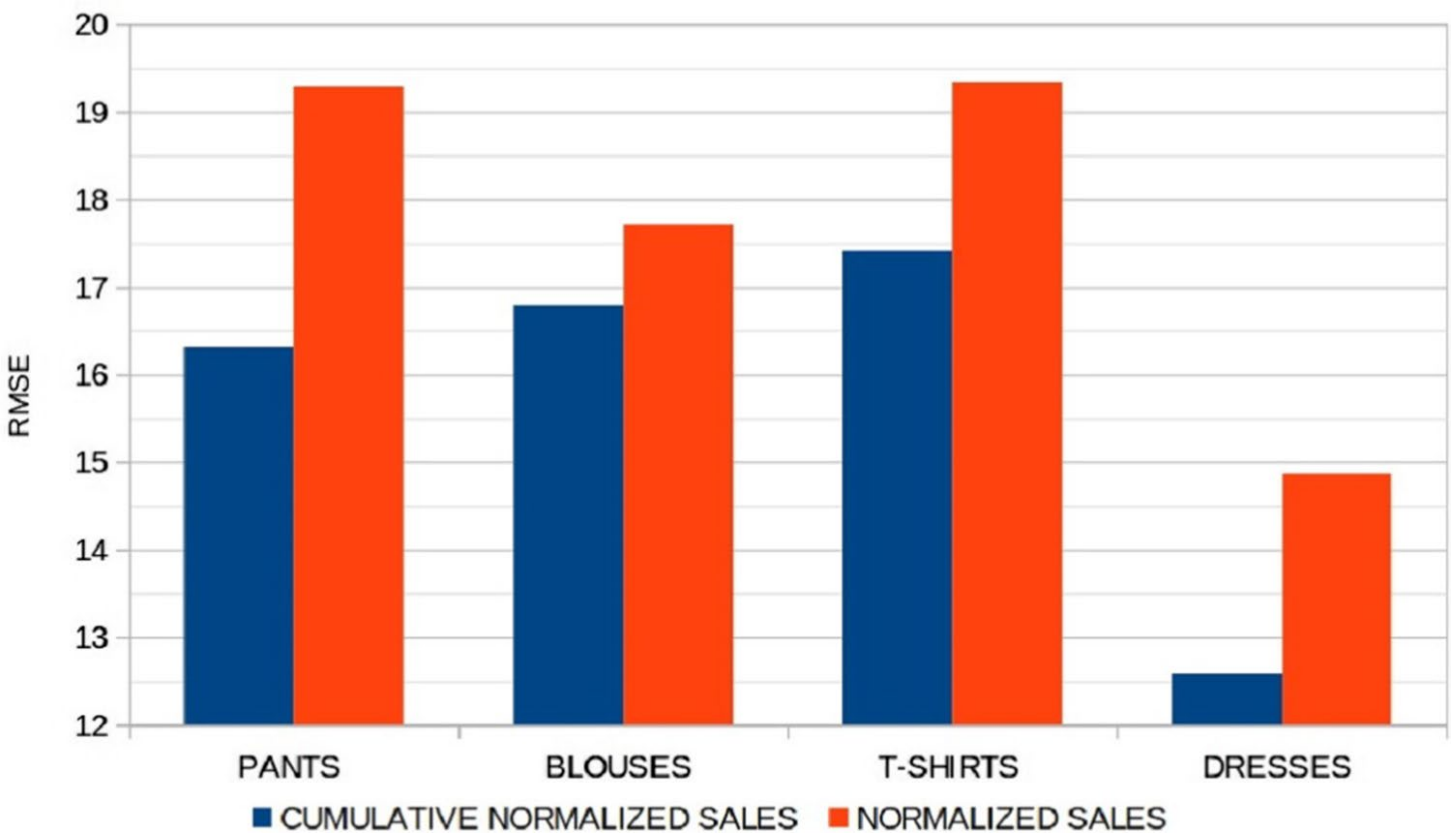
Root mean squared errors when applying the proposed methodology to normalized and cumulative normalized sales

The clustering obtained has been compared with a more advanced clustering technique based on the “SOM followed by K-means” algorithm (Sect. 5.3). The results are presented in Fig. 9 and show that the errors obtained are similar. This demonstrates that the use of cumulative sales profiles makes clustering easier and more straightforward, so a simple clustering algorithm could yield good results. In addition, we tested our classification methodology (K-means + RF) on normalized sales profiles (not cumulated). Figure 10 shows that cumulative sales enable lower forecasting errors for the different datasets used. In fact, as seen in Fig. 6, using cumulative sales avoids having to identify overlapping clusters.

To conclude, we were able to achieve an efficient and robust estimation of different sales patterns using K-means clustering based on cumulative sales profiles.

#### Classification of New Products

**Fig. 11:**
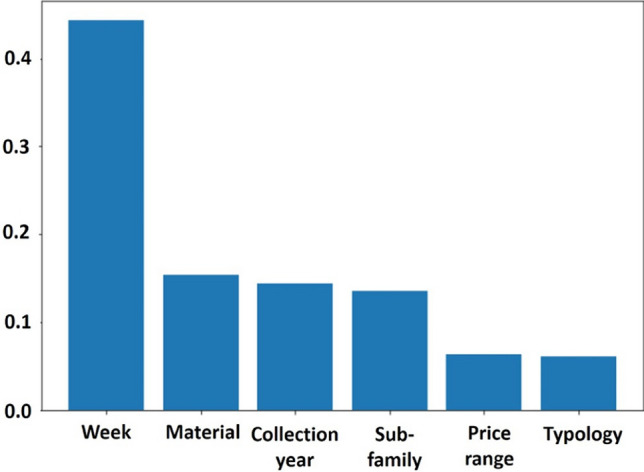
Features importance for the Pants’ dataset

The assignment of the corresponding sales profile cluster, or sales prototype, to each new item, is performed from the descriptive criteria of products (see Sect. 5.1). The RF classifier is learned from the historical products (2019 datasets) using the descriptive criteria, as inputs, and the label of sales profile clusters, as output. The learning process is based on 10-fold cross-validation along with a grid search for tuning the model’s parameters. Table 2 presents the tuned parameters of our final models. In order to get a more robust classification model, a selection of the most relevant attributes should be performed. During the learning of a RF classifier, the features’ importance in the model construction can be quantified. To this end, we use the “Gini Importance” or “Mean Decrease in Impurity (MDI)” algorithm which is defined as the total decrease in node impurity averaged over all trees of the ensemble—it sums up the gain associated with all splits performed along a given variable (improvement in squared error). From the MDI of all available features, only features that have the most important contribution in building the model (having the highest MDI) were selected for the classification task. Figure 11 shows an example of the features’ importance obtained for the pants dataset. Obviously, the week of the first sales is the most important feature considered by the RF. However, the material of the product as well as its sub-category also play an important role in the classification phase, whereas the price range and the product typology can be considered irrelevant and deleted.

**Table 2:** Optimal parameters of the final random forest models for the four datasets

	Maximum depth	Number of trees
Pants	7	100
Blouses	6	150
T-shirts	6	150
Dresses	8	100

As mentioned in Sect. 5.3, three other classification algorithms were tested for comparison with the RF classifier: NB, SVM and MLP. The classification accuracy obtained for each of the datasets as well as the overall forecasting errors for the different tested classifiers are presented in Tables 3 and 4 respectively. Results indicate that, for the different datasets used, the RF classifier outperforms other classifiers with a higher classification accuracy and a lower forecasting error. Moreover, those results reflect the ability of the proposed system to efficiently perform long-term sales forecasting of new products. The lowest classification accuracy (62.25%) is obtained for the T-shirts dataset. This may be due to the fact that the features used to perform the classification is not rich enough for this family of products to be able to yield higher classification accuracy.

**Table 3:** Classification accuracies of the different classifiers

	Pants (%)	Blouses (%)	T-shirts (%)	Dresses (%)
NB	60.85	67.21	57.54	82.08
SVM	61.67	68.43	55.13	81.41
MLP	56.20	60.03	53.65	70.75
RF	**70.15**	**71.15**	** 62.25**	**83.22**

Highest accuracies are shown in bold

**Table 4:** Root mean squared errors when using different classifiers

	Pants	Blouses	T-shirts	Dresses
NB	18.60	17.44	18.76	13.02
SVM	17.84	17.42	18.99	13.36
MLP	19.74	18.89	19.78	15.89
RF	**16.31**	**16.80**	**17.41**	**12.58**

Lowest errors are shown in bold

The sales prototypes predicted with the RF classifier represent the sales profiles forecasting of the items of the year 2020 performed in a regular environment (without COVID-19 crisis). From the real sales of the new products for the first 6 weeks of 2020 (before the beginning of the COVID-19 crisis) (see Sect. 3) and the predicted sales profiles, the predicted sales quantities for each product are then computed for the whole period (January–July). These sales forecasting should be the final predictions for 2020 if the COVID-19 crisis had not occurred.

### Sales Profiles Correction

The sales profiles of new items predicted with the model described in Sect. 6.1 are suitable until week 6 of the year 2020. Then, sales are deeply disturbed and the forecasting model becomes unusable. The proposed methodology consists of predicting sales from week 18 (stores’ re-opening) based on:

- the sales profile forecasting obtained in regular conditions (Sect. 6.1), which still contains important information on seasonality, trends, etc., related to the products,
- a corrective model trained with the real sales recorded till week 17 which reflects the resilience of consumer behavior (Sect. 3).

Obtaining accurate forecasts from week 17 is important to our fashion retailer partner since it is the beginning of stores’ re-opening and there is a crucial need to manage the supply chain: knowing which items of the previous collection must be kept and which new items must be supplied to stores.

As described in Sect. 4.3.3, a regression model is defined from sales of weeks between 13 and 17 to quantify the crisis impact on sales and correct the predictions obtained in a regular environment. A two-step process is implemented for the learning of the regression model:

- the type of regression model (multiplicative or additive coefficient) and the regression order are determined with a cross-validation process on the dataset composed of all the products of the same cluster,
- then, a fine-tuning of coefficients of the regression model is performed for each product data.

The outputs of these regression models are the corrective (multiplicative or additive) coefficients of the sales profiles for weeks after week 17.

**Fig. 12:**
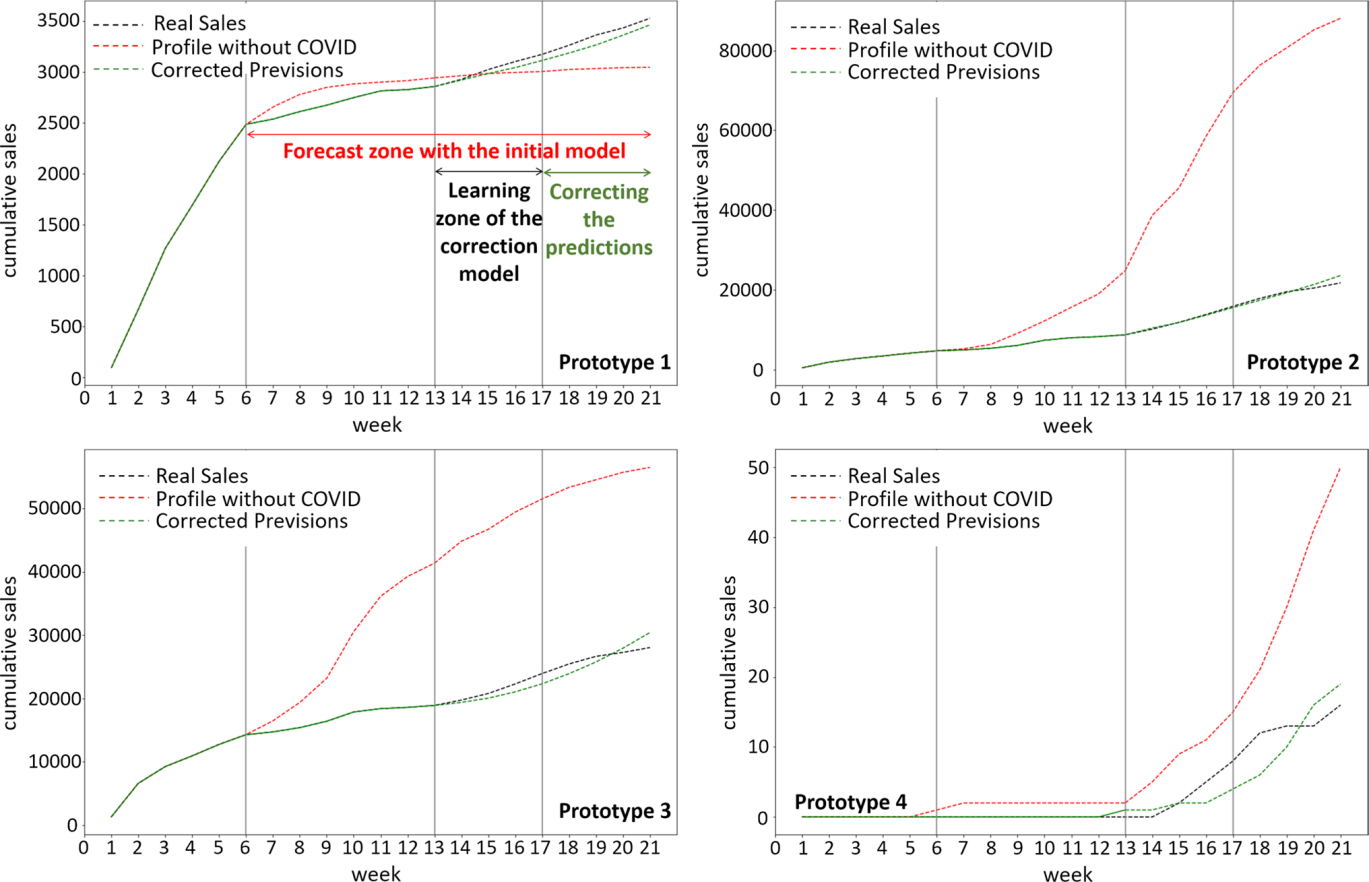
The aggregation of cumulative sales of the blouses’ family, for the year 2020, without and with the impact of the sanitary crisis

Table 5 shows the RMSE obtained by cross-validation on the four prototypes of the blouses. The results show that the regression order significantly affects the accuracy for the two types of regression, multiplicative and additive. For example, the zero-order regression always gives the highest errors, which implies that the correction of the sales prototypes is time-dependent: the impact of the crisis on the sales changes over time. The lowest errors were obtained by a first or second-order regression; no order greater than two was tested in order to avoid overfitting. An example of the final prediction results obtained for the blouses family is shown in Fig. 12.

**Table 5:** Root mean squared errors computed on validation data

	Multiplicative coefficient order 0	Multiplicative coefficient order 1	Multiplicative coefficient order 2	Additive coefficient order 0	Additive coefficient order 1	Additive coefficient order 2
Prototype 1	0.326	0.294	**0.228**	0.253	0.294	0.285
Prototype 2	0.132	0.123	0.116	0.135	0.123	**0.114**
Prototype 3	0.214	0.197	0.193	0.249	0.204	**0.189**
Prototype 4	0.148	0.095	0.108	0.149	**0.092**	0.106

Lowest errors are shown in bold

The analysis of Fig. 12 highlights the impact of the crisis on sales after week 6, especially for the second and third sales profile clusters (top right, bottom left). In addition, for the first prototype (top left), no important change is noticed in the sales behavior, especially because the items of this cluster are mainly sold during the first weeks (weeks 1–6). In fact, for almost all the sales prototypes, the regression model used was able to adjust the sales profile efficiently. This is clearly shown by comparing the predicted sales without a crisis (red curve) and the corrected previsions (green curve) with the real sales (black curve) that actually happened for weeks between 17 and 21. However, for the fourth prototype, the correction model did not perform as well as for the other three prototypes. This may be due to the low number of products assigned for this prototype.

Table 6 presents the root mean squared errors between concatenated real sales and predictions (initial and corrected) between weeks 17 and 21 for the different families of products. Results showed that for all families of products, our proposed methodology of correcting sales predictions enables a significant reduction of forecasting errors. The proposed methodology has been very effective in correcting the initial forecasts and has significantly reduced the root mean squared errors between real and predicted (corrected) sales.

**Table 6:** Root mean squared errors between actual and predicted (initial and corrected) sales

	Initial predictions	Corrected predictions
*Pants*		
Prototype 1	128	**22**
Prototype 2	18060	**150**
Prototype 3	598	**407**
Prototype 4	2209	**173**
*Blouses*		
Prototype 1	340	**74**
Prototype 2	28035	**1540**
Prototype 3	60997	**955**
Prototype 4	22	**4**
*T-shirts*		
Prototype 1	2782	**1253**
Prototype 2	53844	**1493**
Prototype 3	75363	**1964**
Prototype 4	1042	**46**
*Dresses*		
Prototype 1	634	**276**
Prototype 2	6727	**255**
Prototype 3	3461	**1205**
Prototype 4	153	**81**

Lowest errors are shown in bold

## Limitations and Perspectives

Despite the interesting results obtained, this work is subject to several limitations. Firstly, in order to be able to quantify the impact of the pandemic on sales, the proposed methodology relies on the online sales of the company to capture consumer behavior. It cannot be implemented if a company has no sales channel available during the lockdown (for instance a company with only physical stores). Secondly, analyzing and understanding consumer behavior is crucial when designing the sales correction model. Thus, the proposed methodology is limited to B2C—business-to-consumer—companies and the consumer goods market. Finally, the proposed model is only designed to perform short or mid-term predictions. In fact, the impact of a crisis on consumers’ behaviors may diminish over time, making our model not suitable for a long-term prediction.

Future work can be conducted in order to improve the proposed model. First, applying it to other types of crisis, such as a geopolitical crisis, can be done to demonstrate its ability to be generalized and applied to other domains. Secondly, applying the proposed model to other types of goods, for which more historical data can be obtained, allows improving the system by having the possibility of using more advanced machine learning techniques, such as deep learning, which may improve the overall system’s efficiency. Explainable artificial intelligence models can also be implemented, allowing a better understanding of the AI-decision making process which is crucial for any organization. Moreover, additional and different features can be added to the classification task, for each family of products, so that the classification accuracy can be increased, yielding lower forecasting errors. Furthermore, integrating social sciences concepts into the model, with the help of experts in such fields, can yield a better knowledge and understanding of consumers and market behaviors. In fact, by building a correction model that further imitates consumers’ behaviors, the proposed methodology could be generalized and applied in other activity sectors, where sudden and brutal events (pandemics, natural disasters, major social movements, etc.) occur and traditional forecasting systems could no more yield accurate predictions.

## Conclusion

The fashion industry is one of the economic sectors that were seriously impacted by the COVID-19 pandemic. Existing sales forecasting systems have become inapplicable due to the remarkable change in consumer purchasing behaviors, which has left retailers in a situation where they have to rapidly act to deal with the new environment’s constraints. Motivated by a real business project, this study proposed a new sales forecasting methodology that can be applied in such an exceptional context—the COVID-19 pandemic, where train and test datasets present different constraints. Our proposed methodology suggests starting with a normal, yet advanced, sales forecasting system as if there is no additional constraint for the test set—2020’s sales. For this purpose, we used a clustering procedure, applied to cumulative sales profiles, that aims to identify k different sales prototypes, followed by a RF classifier that assigns the corresponding sales prototype for each new item based on its characteristics. Thereafter, once normal predictions are obtained, a regression approach is proposed to model the crisis’ impact on sales and correct the predictions accordingly. The proposed correction model is optimized within the time window made up of the few weeks following the start of the crisis, where real sales (affected by the pandemic) and previously predicted sales are available. By analyzing sales behaviors in this window, and optimizing the regression models’ parameters, the proposed model is able to predict an optimal correcting coefficient capable of correcting the predicted sales for the next weeks (short-term prediction).

We tested the proposed methodology on four different datasets of online sales corresponding to four different families of products. The results showed that we were able to correct sales predictions on the desired period (four weeks) in an efficient way for all of the datasets. Hence, our proposed framework has offered an effective tool to our fashion retailer partner to better manage his supply chain, by knowing which items from the previous collection must be kept in stores, and which new items must be supplied to stores after the end of the lockdown period.

## Data Availability

The data used in this study are real data coming from a private company. Data are confidential and can not be shared.

## Abbreviations

CBR: Case-Based Reasoning
MDI: Mean Decrease in Impurity
ML: Machine Learning
MLP: Multi-Layer Perceptron
NB: Naïve Bayes
PNN: Probabilistic Neural Network
RF: Random Forest
RMSE: Root Mean Square Error
SKU: Stock-Keeping Unit
SOM: Self-Organizing Map
SVM: Support Vector Machine

## Code Availability

This work is based on a custom code from real data of a private company. It can not be shared.
